# Recent advances in the management of Wilms' tumor

**DOI:** 10.12688/f1000research.10760.1

**Published:** 2017-05-12

**Authors:** Roberto I. Lopes, Armando Lorenzo

**Affiliations:** 1 Division of Urology, The Hospital for Sick Children, Toronto, ON, Canada; 2 Department of Surgery, University of Toronto, Toronto, ON, Canada

**Keywords:** Wilms' tumor, nephroblastoma, surgery, chemotherapy, radiotherapy

## Abstract

The objective of this article is to present an overview of recent trends in the management of Wilms’ tumor. With improved survival rates in the past few decades, critical long-term adverse therapy effects (such as renal insufficiency, secondary malignancies, and heart failure) and prevention measures (i.e. nephron-sparing surgery and minimizing the use of radiotherapy) have gained worldwide attention. Specific disease biomarkers that could help stratify high-risk from low-risk patients, and therefore fine-tune management, are in great demand. Ultimately, we aim to enhance clinical outcomes and maintain or improve current survival rates while avoiding undesirable treatment side effects and minimizing the exposure and intensity of chemotherapy and radiotherapy.

## Introduction

Wilms’ tumor (WT), or nephroblastoma, is the most common genitourinary malignant tumor in children. The incidence in the United States is approximately seven new cases per million children, with a peak incidence between 2 and 3 years of age ^1^. Clinically, WT typically presents as an asymptomatic abdominal mass, which is felt by the parents or caretakers in most cases. Gross hematuria, abdominal pain, or hypertension can be observed in up to a quarter of patients ^1^.

Abdominal ultrasound (US) examination is the initial imaging study of choice, as it confirms the presence of a renal mass without ionizing radiation and gives a preliminary assessment of the contralateral kidney as well as the presence of metastatic disease and the presence of a tumor thrombus. However, magnetic resonance imaging (MRI) or computerized tomography (CT) are key tests to obtain in order to gain all necessary information for diagnosis and staging. Chest CT is the standard modality for thoracic metastatic assessment ^1– 3^.

Surgery, chemotherapy, and, in some patients, radiotherapy comprise the treatment for WT. The initial management follows one of two treatment protocols: one that is recommended by the Children’s Oncology Group (COG), and another that is recommended by the Société Internationale d’Oncologie Pédiatrique (SIOP). Because of differences in treatment philosophy, the staging systems followed by each are somewhat different. These are presented in Table 1. Attention should be directed at the impact of pre-operative biopsy as well as the implications of staging done before or after chemotherapy.

**Table 1.** Children’s Oncology Group (COG) and Société Internationale d’Oncologie Pédiatrique (SIOP) staging systems.

	COG	SIOP
Stage I	Tumor is limited to the kidney and has been completely resected. The tumor was not ruptured or biopsied before removal. No penetration of the renal capsule or involvement of renal sinus vessels.	The tumor is limited to the kidney or surrounded with a fibrous pseudocapsule if outside the normal contours of the kidney and is completely resected. The tumor may be protruding (bulging) into the pelvic system and dipping into the ureter, but it is not infiltrating their walls. The vessels of the renal sinus are not involved. Intrarenal vessels may be involved. Presence of necrotic tumor in the renal sinus or perirenal fat does not upstage to stage II. Percutaneous cutting needle biopsy is allowed.
Stage II	Tumor extends beyond the capsule of the kidney but was completely resected with no evidence of tumor at or beyond the margins of resection. There is penetration of the renal capsule or invasion of the renal sinus vessels.	The tumor extends beyond the kidney or penetrates through the renal capsule and/or fibrous pseudocapsule into the perirenal fat but is completely resected. The tumor infiltrates the renal sinus and/or invades blood and lymphatic vessels outside the renal parenchyma, but it is completely resected en bloc. The tumor infiltrates adjacent organs or the vena cava but is completely resected. Percutaneous cutting needle biopsy is allowed.
Stage III	Gross or microscopic residual tumor remains post- operatively including inoperable tumor, positive surgical margins, tumor spillage surfaces, regional lymph node metastases, positive peritoneal cytology, or transected tumor thrombus. The tumor was ruptured or biopsied before removal.	Incomplete excision of the tumor, which extends beyond resection margins (gross or microscopic tumor remains post-operatively). Any abdominal lymph nodes are involved. Tumor rupture before or during surgery (irrespective of other criteria for staging). The tumor has penetrated the peritoneal surface. Tumor implants are found on the peritoneal surface. The tumor thrombi present at resection, margins of vessels, or ureter are transected or removed piecemeal by the surgeon. The tumor has been surgically biopsied (wedge or open biopsy) prior to pre-operative chemotherapy or surgery.
Stage IV	Hematogenous metastases or lymph node metastases outside the abdomen (e.g. lung, liver, bone, and brain).	Hematogenous metastases (lung, liver, bone, brain, etc.) or lymph node metastases outside the abdominopelvic region.
Stage V	Bilateral renal involvement is present at diagnosis.	Bilateral renal tumors at diagnosis. Each side has to be substaged according to the above classifications.

### Children’s Oncology Group

The COG advocates for up-front surgical removal without the administration of neoadjuvant antineoplastic drugs, thus providing a detailed histological staging and accurate molecular DNA-based studies (i.e. loss of heterozygosity [LOH] test), which are necessary for planning post-operative treatment. There are, however, some important exceptions to this approach. For COG protocols, neoadjuvant chemotherapy is recommended in cases with tumor thrombus extending above the level of the hepatic veins, gross involvement of contiguous structures whereby the only means of removing the tumor requires removal of the kidney (with exception of the adrenal gland), bilateral WT, extensive pulmonary compromise from a compression by a massive tumor or widespread metastatic disease, and when, according to the surgeon’s discretion, immediate nephrectomy would result in significant morbidity, including tumor spill or incomplete resection ^2, 4^.

There are potential disadvantages to pre-nephrectomy chemotherapy. These include loss of staging information (such as eradicating neoplastic cells from lymph nodes), treatment of a benign condition with chemotherapy or initiating treatment of a different malignant disease with a potentially inappropriate chemotherapy protocol, tumor growth during treatment (potentially making surgical resection more difficult), and tumor rupture while receiving chemotherapy and waiting for surgery ^4^. Previous trials have suggested that up to 5–10% of patients with the pre-nephrectomy diagnosis of WT have a benign or malignant condition other than WT ^5^. In recent years, this figure has been called into question and may be as low as 1% ^6^. SIOP generally recommends avoiding biopsy; it may be conducted in a minority of cases, usually represented by doubtful cases such as unexpected age range group, radiological atypical characteristics, and/or poor chemotherapy response.

The information gained by up-front resection (COG) allows for histologic classification that separates tumors into two broad categories based on the presence or absence of adverse pathological features: favorable histology (no anaplasia) versus unfavorable histology (focal anaplasia or diffuse anaplasia). In addition, extension outside of the kidney, invasion of vessels or perinephric tissue, and presence of lymph nodes with metastatic disease add data to complete staging based on COG protocols.

### Société Internationale d’Oncologie Pédiatrique

SIOP advocates for a standard chemotherapy protocol before nephrectomy, even in the absence of metastatic disease or presence of a large tumor thrombus. Despite the potential adverse effects of neoadjuvant chemotherapy in staging and histologic evaluation, its benefits include the personalized *in vivo* assessment of histological response to chemotherapy, including the identification of a “high-risk” category of blastemal-type WT. Often it also leads to a reduction in tumor size and the formation of a fibrous pseudocapsule that facilitates surgical removal and decreases the risks of rupture and spillage during surgery (thus obviating the need for radiotherapy) ^7–11^. Pre-nephrectomy chemotherapy has also been shown to decrease the risk of intra-operative hemorrhage ^2^.

Up-front nephrectomy is recommended for renal tumors in children younger than 6 months old, as congenital mesoblastic nephroma is more prevalent in this age group and does not require chemotherapy for its treatment ^12^. Malignant rhabdoid tumor of the kidney may also present at this young age and is treated with different chemotherapy to that used for WT. Unusual presentations of renal tumors usually require biopsy to confirm WT.

Based on the impact of therapy prior to surgery, SIOP also subclassifies WT and risk stratifies based on histologic changes after chemotherapy. Patients are divided into low-, intermediate-, and high-risk groups considering the degree of tumor necrosis and relative proportion of each of the three cellular components (epithelial, stromal, or blastemal). Patients with diffuse anaplastic or blastemal-type WT once chemotherapy has finished are considered to have high-risk histology ^13^.

In both protocols, chemotherapy is based on vincristine (VCR) and dactinomycin (AMD) for stage I and II with favorable histology. VCR, AMD, and doxorubicin (DOX) are used for stage III and IV with favorable histology according to COG; SIOP does not recommend the addition of DOX for stage III favorable (intermediate- or low-risk) tumors ^1, 4, 14^. Advanced stages (II, III, and IV) of tumor with anaplasia or higher-risk tumors demand therapy intensification by introducing other drugs (i.e. cyclophosphamide, ifosfamide, carboplatin, and etoposide) and radiation therapy. Usually, four drugs are selected for this scenario. Stage V disease (bilateral tumors) requires pre-operative chemotherapy with VCR, AMD, and eventually DOX for 6 to 12 weeks, followed by nephron-sparing surgery (NSS). The survival rates for both strategies are similar when taking surgical removal, pre- and/or post-operative chemotherapy, and radiotherapy into consideration. Both protocols now focus on minimizing late effects of treatment without compromising the current excellent overall survival rates ^1, 4^. In addition, both cooperative groups are in search of better treatment options for patients with adverse features and suboptimal survival rates.

## Surgery

The current standard surgical procedure for a unilateral WT is a transperitoneal radical nephroureterectomy with ipsilateral lymph node sampling ^1– 3^. Although extensive lymphadenectomy is not required, perihilar and periaortic or pericaval lymph node samples must be obtained in all cases, as they are necessary for adequate staging and planning of post-operative management ^2, 3^.

Bilateral WT (BWT) affects approximately 5% of children ^1^. The presence of BWT should raise suspicion for predisposition syndromes (such as WAGR, Denys-Drash, and Beckwith-Wiedemann). Similarly, this should be considered in children with multifocal and recurrent tumors as well as an earlier age of onset ^15^. Renal insufficiency occurs in a much greater proportion than in children with BWTs when compared to unilateral tumors (10% versus 0.7%) ^1^.

For BWTs, neoadjuvant chemotherapy is recommended for 6–12 weeks (in both COG and SIOP protocols). Chemotherapy is dictated by the individual response, with tumors reimaged to assess response after the second cycle of chemotherapy. As a general guideline, if there is poor (less than 50%) response, bilateral biopsies should be performed to determine whether there are anaplastic elements or rhabdomyomatous changes. In patients who exhibit a favorable response to chemotherapy, two extra cycles of chemotherapy are administered before surgical resection is performed ^1, 3^.

A Chevron or transverse incision is generally used. Care must be taken to completely expose the kidney and tumor on both sides and to identify mesenteric and renal vessels to prevent inadvertent damage. Possible alternatives are bilateral partial nephrectomy or unilateral radical nephrectomy (RN) and contralateral partial nephrectomy. As shown by Davidoff *et al*., most cases are amenable to NSS ^16^.

## Adjuvant chemotherapy and/or radiotherapy

Post-operative treatment depends on the stage of the disease as well as the histological evaluation of the specimen and the lymph nodes obtained in surgery. Adjuvant chemotherapy is recommended for all tumor stages after immediate nephrectomy, with the exception of children younger than 2 years of age with stage I disease and tumors with favorable histology weighing <550 g ^1, 3^. Stage III patients or patients with anaplastic histology receive local irradiation according to COG, meanwhile SIOP recommends local radiation therapy (XRT) for stages II and III with anaplasia ^1, 3^. A major tumor rupture requires whole abdominal XRT ^1^.

## Long-term treatment effects

Although significant success has been achieved by increasing the overall 5-year survival rates to more than 90%, multimodal therapy is associated with late adverse effects, which require long-term monitoring of these patients ^17– 19^. The standardized mortality ratio (SMR) is 24.3 during the first 5 years after diagnosis but remains increased for more than 20 years afterwards (SMR 4.3) ^17, 18^. Although initially morbidity and mortality are driven by the neoplastic process, as patients survive, other factors are at play.

Around 0.7% of patients with unilateral WT will develop renal failure. Although a relatively small figure, it reflects an incidence that is eightfold higher than that expected in the age-matched general population ^17, 18^. Patients who previously underwent a RN are at risk of presenting a trend towards progressive decline in eGFR with aging ^20, 21^. Conversely, patients who underwent NSS appear to have a more favorable trend with preserved eGFR up to the third decade of life ^20– 23^.

The cumulative incidence of a second malignancy is 1.6% at 15 years from diagnosis; abdominal radiotherapy as part of the initial or adjuvant therapy is among the risk factors ^18, 19^. Secondary sarcomas are some of the most common SMN. The risk of developing a secondary leukemia following WT therapy has been estimated at 0.2% at 25 years and remains stable at 30 years of follow-up ^18, 19^.

Cumulative incidence of congestive heart failure (CHF) 20 years after diagnosis of WT is approximately 4% in patients whose treatment plan included DOX, with a direct dose-response relationship (each 100 mg/m ^2^ of DOX exposure increased the relative risk of CHF by 3.3) ^17, 18^.

## Surveillance

Surveillance should be offered to children at increased risk (>5% risk of WT) ^24^. A clinical geneticist must be involved, and renal US should be carried out every 3 months. Surveillance is recommended to continue until 5 years of age in predisposition due to *WT1* mutant syndromes, and until at least 7 years of age in Beckwith–Wiedemann syndrome, isolated hemi-hypertrophy with underlying 11p15 imprinting disruption at the IGF2/H19 locus, and in familial WT pedigrees ^24^.

## Metastatic and recurrent disease

WT most commonly spreads to the lungs and the liver. Patients who have hematogenous metastatic disease of the lung, liver, or other areas are classified by both the SIOP and the COG staging systems as having stage IV disease, irrespective of local tumor stage.

With the SIOP approach, 6 weeks of a three-drug regimen (VCR, AMD, and DOX) chemotherapy before nephrectomy is advised for patients with lung metastasis at diagnosis ^25^. If a complete response is observed or lung nodules are completely surgically resected, patients do not receive lung XRT ^23^. Chemotherapy after the initial 6 weeks is based on histologic findings: most patients with intermediate-risk disease with a good response continue three-drug chemotherapy with a cumulative DOX dosage of 250 mg/m ^2^ in the upcoming UMBRELLA protocol (SIOP). For poorer responders, a four-drug regimen (300 mg/m ^2^) is recommended ^2, 12, 25^.

COG (AREN0533 study) adapts therapy according to lung nodule response ^2, 25^; 6 weeks of treatment with VCR/AMD/DOX is administered. If a complete response occurs, patients continue the same chemotherapy with a cumulative DOX dose of 150 mg/m ^2^ and lung XRT is omitted. If it is not a complete response, biopsy is advisable. If the lung nodule(s) are confirmed to be tumors or if they were not biopsied, patients receive lung XRT. This augmentation of therapy apparently suggests improved outcomes for patients with incomplete lung nodule response. The avoidance of lung XRT in select patients with stage IV WT represents a common pathway between COG and SIOP ^25^.

Liver metastases are similar to pulmonary nodules in that they do not require up-front operation and that most can be treated with chemotherapy and radiotherapy ^26^.

Recurrent disease is difficult to treat in children with WTs with only ~50% survival ^12^. Most recurrences happen by 18 months. The tumor bed and/or the lungs are the two most frequent sites of recurrence ^2, 27, 28^.

## Minimally invasive surgery

Minimally invasive surgery (MIS) for WT treatment should be considered for selected patients and in centers with experience and documented outcomes, as it is difficult to reliably obtain negative margins or avoid rupture, which can increase the risk of local recurrence, adversely impact survival, and demand therapy intensification ^29^.

**Figure 1.**
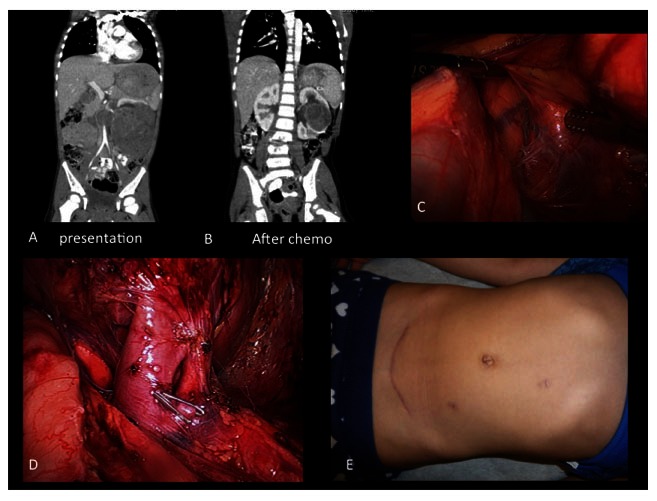
Laparoscopic nephrectomy after chemotherapy. **A**. Large left renal mass; **B**. shrinkage of the tumor after chemotherapy, although it is still not amenable for a partial nephrectomy; **C**. left laparoscopic transperitoneal radical nephrectomy; **D**. dissection of renal artery and vein; **E**. final cosmetic result.

An open RN with lymph node sampling is still recommended and favored for the surgical treatment of most unilateral WTs. However, current evidence suggests that, in experienced hands and selected cases, laparoscopic transperitoneal nephrectomy may offer the same outcome as the classical open approach ^3, 30^. Figure 1 shows some of the steps of a radical transperitoneal laparoscopic nephrectomy.

## Nephron-sparing surgery

NSS has been advocated to decrease the risk of late renal failure ^22, 23^. The major drawbacks of a partial nephrectomy for WT are a theoretical risk of tumor spill and/or a positive surgical margin, necessitating abdominal irradiation and possibly the use of DOX in addition to VCR and AMD ^3, 29^.

A systematic review on more than 4,000 WTs showed similar rupture rates between RN and NSS (13% versus 7%), as well as recurrence rates (12% versus 11%) and survival rates (85% versus 88%) ^31^. Using Surveillance, Epidemiology, and End Results data (from 1998 to 2010), Wang *et al*. evaluated 876 boys and 956 girls with WT (mean age 3.3 ± 2.9 years) ^17^. Of these patients, 114 (6.2%) underwent NSS (unilateral WT in 74 and bilateral in 37). Median follow-up was 7.1 years. NSS was associated with smaller, bilateral tumors and with omission of lymphadenectomy. Despite lymph node under staging, overall survival was similar between patients undergoing NSS and RN ^17^.

The prospective SIOP WT 2001 study (with 2,800 patients) showed a clear over-representation in patients undergoing NSS of smaller tumors and with a more favorable stage and size distribution ^32^. In 2,709 patients, a total nephrectomy (TN) was performed, and 91 (3%) underwent NSS. The NSS group contained more stage I tumors (65% versus 48%) and fewer stage III tumors (13% versus 26%) compared to the TN group ( *p*=0.0005). Additionally, tumor volumes were smaller in the NSS group ( *p*<0.001). No differences in ruptures of tumor capsule or lymph node ruptures were observed. Event-free and overall survival were similar in both groups. The recommendation by the group was that, despite these favorable observations, the consideration of NSS still needs to be carefully weighed on a case-by-case basis against the potential risk of inducing stage III disease with the consequence of abdominal radiotherapy. The SIOP 2001 protocol dictates radiotherapy be administered to the tumor bed in case of positive margins, positive lymph nodes, and/or tumor spill ^32^.

**Figure 2.**
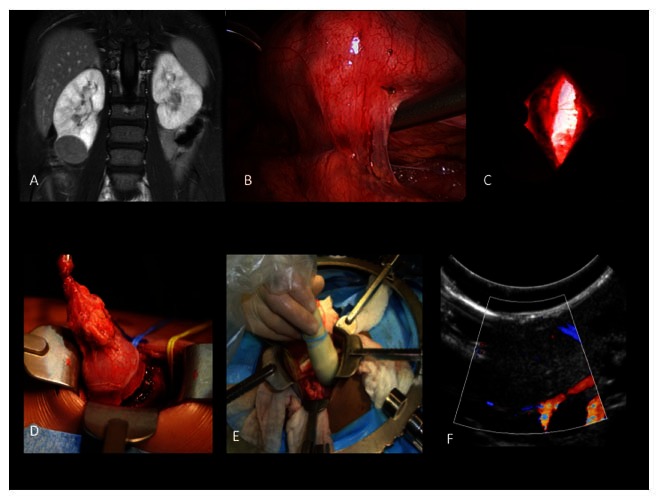
Zero-ischemia laparoscopic assisted open partial nephrectomy. **A**. Lower pole right renal tumor; **B**. laparoscopic mobilization of the kidney, especially the lower pole tumor; **C**. subcostal incision guided by laparoscopy (light coming from the abdomen); **D**. mobilization of the lower pole tumor (reducing risks of tumor spill)—note the presence of vessel loops placed laparoscopically (as a safety resource for bleeding control); **E**. intra-operative ultrasonography helps define the tumor and aids the identification of a margin-free resection; **F**. ultrasonographic view of the tumor.

The recently published technique by our group—“zero-ischemia” laparoscopic-assisted partial nephrectomy—is illustrated in Figure 2 ^29^. This strategy allows a safe NSS for selected cases with acceptable morbidity and potentially better cosmesis and recovery than the traditional open approach.

## Biomarkers of disease

The *WT1* gene, located at chromosome 11p13, was one of the first tumor suppressor genes described in WT ^33^. Consequently, *CTNNB1* and *WTX* have been identified in tumors. *WT1*, *CTNNB1*, ** and *WTX* genetic alterations are estimated to co-occur in approximately one-third of WTs ^34^. Many other genes appear to be implicated, including *TP53* and *MYNC* ^34, 35^.

The use of LOH assays to determine areas of allele loss has shown that the majority of WTs have few or no changes and that these tend to be restricted to a few loci, principally at 1p, 11p, 11q, 16q, and 22q ^34^. COG/NWTS has reported that LOH involving 1p and 16q correlate positively with a poorer prognosis, which has triggered recommendations for a modest increase in intensity of drug regimens ^36^. Other studies have also revealed an association of LOH at chromosomes 1p, 11q, 16q, and 22q with an increased risk of relapse ^36^. Gain of 1q has also been recently reported as a potentially important prognostic biomarker in WT ^37, 38^.

## Recent systematic reviews and meta-analyses

Based on a recent meta-analysis, the pooled prevalence of *WT1, WTX*, and *CTNNB1* somatic mutation in patients with WT was 0.141 (0.104, 0.178), 0.147 (0.110, 0.184), and 0.140 (0.100, 0.190), respectively ^34^. The incidence of *WT1* and *CTNNB1* combined was 28.1%, and *WT1* and *WTX* combined was 28.8%.

Accumulation of the TP53 protein in WT specimens has been associated with unfavorable histology and treatment resistance ^35^. There is a clear relationship between *TP53* mutations and anaplastic WT ^35^. This indicates that these mutations are related to tumor progression and associated with a more aggressive type of disease. In anaplastic WT, the pooled frequency of *TP53* mutation was 0.410 (0.214, 0.605) ^34^. This indicates that testing for such alterations may be advisable, especially if there is any hint of anaplasia.

A recent systematic review evaluated a total of 40 studies examining 32 biomarkers in 7,381 patients with WT ^39^. The strongest negative prognostic association was LOH at 11p15, with a risk of recurrence of 5, although LOH at 1p and 16q and gain of function at 1q were also strongly linked to increased recurrence (2.93, 1.95, and 2.86, respectively) ^39^. A limitation of LOH at 1p/16q as a biomarker is the relatively low prevalence, estimated at 4.6% of patients in NWTS and in only 9.4% of recurrences ^36^. A marker that appears to have higher comparative prevalence is gain of function at 1q, which was present in 27% of patients ^37, 38^.

## MicroRNAs

MicroRNAs (miRNAs) are small (~22 nucleotides in length), non-coding RNAs that negatively regulate gene expression at the post-transcriptional level ^38^. Primary miRNAs are transcribed by RNA polymerase II and are subsequently turned into precursor miRNAs (pre-miRNA). Dicer then processes pre-miRNAs into mature miRNAs that are incorporated into the RNA-induced silencing complex (RISC) to mediate the cleavage or translational inhibition of target messenger RNAs (mRNAs). MicroRNAs are involved in many biological processes, such as development, growth, and metabolism. It is also being increasingly demonstrated that miRNAs are dysregulated in WT, suggesting that miRNAs may be important in WT pathogenesis ^40– 42^.

## Upregulated microRNAs

### Oncomir-1

Oncomir-1 is an oncogenic cluster of miRNAs located on chromosome 13, and its upregulation could promote cell proliferation in tumors ^41^. E2F3 acts as a transcriptional activator and is overexpressed in a number of different cancers. miRNA expression profiling demonstrated that oncomir-1 family members, such as miR-92, miR-17-5p, and miR-20a, were upregulated in WT as compared with healthy kidney tissues and other renal cancer types. Atypical activation of the E2F3–oncomir-1 axis may contribute to oncogenesis in WT ^41^.

### MiR-483-3p/5p

Human miR-483 is embedded within the second intron of *IGF2*, which encodes insulin growth factor 2 (IGF2). IGF2, a fetal growth factor, has been shown to increase cell proliferation and promote tumor development and is aberrantly increased in WT. MiR-483-3p is upregulated in 100% of WTs, and a functional positive feedback loop between miR-483-5p and IGF2 has been suggested ^40^.

## Down-regulated microRNAs

### MiR-204

Meis homeobox 1 (MEIS1) is a HOX class protein cofactor and is involved in the regulation of embryonic growth and differentiation. MEIS1 was upregulated in a model of WT. As a predicted upstream regulator of MEIS1, MiRNA-204 expression was significantly decreased in all WT samples compared with normal renal parenchyma ^40^.

### MiR-185

The SIX1 homeobox protein plays a significant role during development, and it has been shown to be the direct target of miR-185 and exhibit an inverse correlation with miR-185 in WT and normal matched control kidney tissues ^40^.

## Circulating tumor DNA

Circulating tumor DNA (ctDNA) can potentially be utilized in the molecular diagnosis of cancer. It makes up a portion of cell-free DNA (cfDNA) depending on the pathology, tumor load, tumor spread, or necrosis ^43^. According to the SIOP, pediatric renal tumors are first regarded as nephroblastomas, with post-nephrectomy histologic confirmation, but other histological diagnoses may follow. In a retrospective study, children with renal tumors and available plasma sample at diagnosis were included ^43^. Extraction of cfDNA was performed and genetic alterations identified in ctDNA were compared to those found in neoplastic and constitutional DNA.

Twenty patients were identified; the median age at diagnosis was 2.1 years. Secondary histologic diagnosis confirmed 17 nephroblastoma cases, two clear-cell sarcomas, and one clear-cell carcinoma. Capillary electrophoresis confirmed the presence of cfDNA in all samples. The study of ctDNA is a promising non-invasive method for the molecular diagnosis/monitoring of tumors still under development ^43^. The identification of specific tumor genetic alterations in ctDNA could be a useful tool to specify the diagnosis of the different renal tumor subtypes, enabling one to guide the up-front management and potentially introduce tumor monitoring during treatment, obviating the need for a biopsy.
